# Supplementary material for: Response time of an avian prey to a simulated hawk attack is slower in darker conditions, but is independent of hawk colour morph
Source: R Soc Open Sci. 2019 Aug 7;6(8):190677. doi: 10.1098/rsos.190677 (PMC6731706; doi:10.1098/rsos.190677)
Supplement: Supplement 1 [file rsos190677supp1.docx]

SUPPLEMENTARY MATERIAL

Nebel C., Sumasgutner P., Pajot A., Amar, A. **Response time of an avian prey to a simulated hawk attack is slower in darker conditions, but is independent of hawk colour morph,** *Royal Society Open Science*

SUPPORTING INFORMATION TO MATERIAL AND METHODS

*Feral pigeons and their housing*

We used 185 individual wild feral pigeons (*Columba livia f. domestica*), these were either caught by ourselves or as part of pest control measures by Eagle Encounters, Stellenbosch, at sites across the Cape peninsula, South Africa. All pigeons were transported to the experimental room and kept overnight in individual cages of 50 x 30 x 30 cm. The experimental trials commenced the next day. While in our care (for a maximum of 24 h, before they were either returned to Eagle Encounters or individually marked and ringed), pigeons had access to water *ad libitum*, but were only fed during the experimental trials the next day, from a stationary feeder fixed in the cage and facing the simulated hawk attack in a standardised fashion. After the experimental days, pigeons were individually marked with cable ties on one of their legs to avoid using recapture and using the same pigeon twice in this experiment.

*Black sparrowhawk mounts*

We used four taxidermy mounted male black sparrowhawks, with two replicates of each morph. The birds were mounted in a flight/attack position (see an example in Figure S1) by a professional taxidermist based in Durbanville, Cape Town, ensuring similar wingspan (67-72 cm), body length (45-48 cm) and weight (250 g). The black sparrowhawks were previously collected during monitoring of the study population on the Cape peninsula, Cape Town, South Africa. The exact causes of their deaths are unknown. The morph of the hawk used first was selected randomly at the start of each trial set and was then alternated to ensure a balanced number of trials using the four mount replicates.

*Experimental set-up and protocol*

A detailed experimental set-up can be seen in Figure 1, which was similar to Cresswell et al (2003, 2009) and Whittingham et al (2004). It consisted of the hawk running down a 12 m-long, 10.3 ° angled fishing line where it comes in sight for the pigeon at a standardised point. The experiment was set-up indoors (room: 15 x 4 x 2.9 m) at the University of Cape Town’s field station in Bainskloof that allows to block all natural light out. In total, the experiment was set up three times with identical measurements. The first two experimental set-ups were done in April and May 2018 (*control-hawk* and *light level-morph* experiment) and a third in June 2018 (*background-morph* and part of the *light level-morph* experiment). The room had only an artificial light source consisting of four ‘redhead’ lamps (Lightstar, Professional Lighting, bulb: Osram, 800 W, 240 V), located above the pigeon cage and angled to light up the ventral side of the on-coming hawk (the side that differs between the two morphs), whilst minimizing glare to the pigeon. The pigeon cage was placed 1 m beneath the line, and a high-speed camera (XDV 4K action camera recording at 90 fps) was mounted in a standard position behind. This camera recorded the moment the hawk came into view and the moment the pigeon responded (Figure S7). Trials were only simulated while the pigeon was feeding to ensure them facing the attacking hawk. The first visibility of the hawk mount for the pigeon and the camera was standardised by a blind which consisted of a square sheet of beige coloured fabric that measured 137 x 120 cm and was located 258 cm from the front of the pigeon cage. Food for the pigeon (a seed mixture for wild garden birds) was placed in a specific food hopper at the front of the cage. After initiating feeding, we waited one minute before releasing the hawk, to prevent the pigeon from associating food with an attack. In case of an unsuccessful trial (i.e. the pigeon would not feed), we aborted the trial after 10 min and would repeat after a minimum 20 min break. 20 min was also the minimum time between every trial.

We created two ambient light treatments: 1) “bright light” conditions, using four lamps on highest intensity, producing 2182 ± 65 lux; and, 2) “dull light” conditions with two middle lamps on highest intensity, which were additionally dimmed by spanning a black fabric across them, producing 112 ± 12 lux (Figure 1). Lux levels were measured at the blind with a digital multimeter MS8229 (Mastech). The same device was used to measure temperature (°C) at the pigeon cage after every trial. Between the first visibility of the hawk and when it passed over the pigeon, the mount covered a distance of 4 m. We played white noise throughout the trials to avoid any sound of the moving hawk mount (70 db white noise, measured at the pigeon cage).

The *light level - morph* experiment, consisted of a complete crossed experiment with four treatment types (dull light–dark morph; dull light–light morph; bright light–dark morph; bright light–light morph) in randomised order. Throughout these trials, we also ran a control to confirm that pigeons recognized the hawk as a threat rather than just responding to moving objects. Within the *control – hawk* experiment, the control was an either pink or blue squared fabric bag (20 x 16 cm) randomly selected and tested on 83.5% of the pigeons and resulting in a maximum of five trials per pigeon. In these trials the background behind the hawk was left white – which was the natural colour of the room.

The *background - morph* experiment, consisted of a complete crossed experiment with four treatment types (black background–dark morph; black background–light morph; white background–dark morph; white background–light morph). Background colour was changed by spanning a black fabric over the ceiling (Figure 1). We did not have the logistical capacity to conduct these treatments in both light conditions, why they were conducted under dull light only. We added a random hawk morph flown under bright light levels to increase the sample size of the *light level - morph* experiment and to have again maximum five trials per pigeon.

During each trial, we measured the time (in seconds (s)) from the moment the hawk came into view until the occurrence of two different responses by the pigeon (a) detection time, which was the initiation of movement of the pigeon to the hawk (in most cases a turn of the head to face the hawk); and (b) reaction time, which was the initiation of an escape or avoidance response (that could range from the pigeon just lowering its body to an escape into the back of the cage). Both measurements are meaningful and represent different cognitive processes: detection time is the detection of an on-coming object, whereas the reaction time will incorporate the detection, the perception of a predatory attack and the decision to respond. In 9.5 % of all trials, we were unable to measure a detection time, because the pigeon was already looking directly in the direction of the hawk. Additionally, we removed trials from the experiment where no escape reaction was recorded (8.8 % of all trials, either because the pigeon missed the hawk – 18.3% - or did not try to escape it – 81.7 %). Time stamps were recorded on the high-speed video in slow motion with the free software MPC-HC 1.7.13. Additionally, we measured the speed of the hawk and control by dividing the distance from the point of view to a fixed-point towards the end of the line by time (m/s).

*Statistical Analysis*

Our analyses used three different linear mixed models (LMM) implemented in the statistical software R, version 1.1.442 (R Core Team 2016) using the ‘*lme4*’ package (v. 1.1-17; Bates et al 2015). The three models tested either detection or reaction time as response variable (log-transformed to fit normal distribution). We controlled for the following fixed covariates: speed of the hawk (mean 2.15 ± 0.28 m/s) or control (mean 2.11 ± 0.26 m/s), head position of the pigeon (either head down at the feeder or looking up) and temperature (°C) in all models. We also fitted one random term, “pigeon ID”, to control for lack of independence of different trials conducted on the same pigeon. We also controlled for hawk replicate, humidity (%), time of the day (h) and number of times the pigeon had seen the hawk (1-5); however, none of these variables were significant and were not included in any of the final models. The final models were chosen by a stepwise backward approach, only including predictors with a significant or trend effect p < 0.1.

Model 1 (*control – hawk* experiment) tested whether response times differed between a hawk model and a control. Model 2 (*light level - morph* experiment) explored whether response times change under different light conditions, and whether this varied depending on the morph of the hawk. Here we used data from all our trials with a hawk mount and a white background: explanatory variables (in addition to afore mentioned fixed covariates) included light level (dull or bright), hawk morph (dark or light) and the interaction between these two terms. Model 3 (*background - morph* experiment) explored whether response times change with different background colours, and whether this varied depending on the morph of the hawk. Here we excluded all trials from the *light level – morph* experiment that were performed under bright light, with the explanatory variables (in addition to speed, head position and temperature) background colour (white or black) together with hawk morph (dark or light) and the interaction between these two. Results are presented in the format mean ± SD with parameter estimates in Table 1.

REFERENCES

Bates, D. M., Maechler, M., Bolker, B. & Walker S. 2015 Fitting Linear Mixed-Effects Models Using lme4. Journal of Statistical Software 67, 1-48 (doi: 10.18637/jss.v067.i01)

Cresswell, W., Quinn, J. L., Whittingham, M. J. & Butler, S. 2003 Good foragers can also be good at detecting predators. Proceedings of the Royal Society of London. Series B: Biological Sciences 270, 1069–1076. (doi:10.1098/rspb.2003.2353)

Cresswell W., Butler, S., Whittingham M.J. & Quinn J.L. 2009 Very short delays prior to escape from potential predators may function efficiently as adaptive risk-assessment periods. Behaviour 146, 795–813. (doi:10.1163/156853909x446217)

R Core Team 2018 R: A Language and Environment for Statistical Computing. R Foundation for Statistical Computing Vienna. <https://www.R-project.org>

Whittingham, M.J., Butler, S. J., Quinn, J. L. & Cresswell, W. 2004 The effect of limited visibility on vigilance behavior and speed of predator detection: implications for the conservation of granivorous passerines. Oikos 106, 377-385. (doi: 10.1111/j.0030-1299.2004.13132.x)


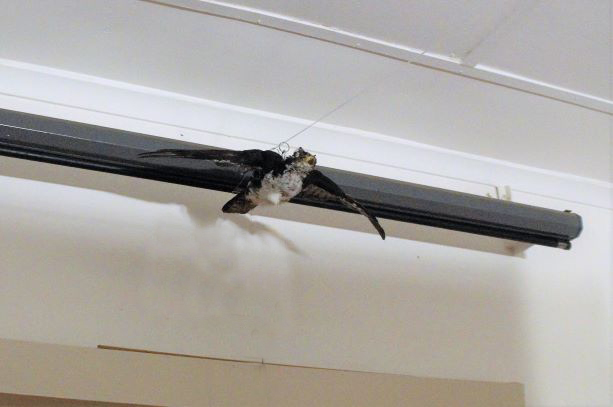


Figure S1. One of the four black sparrowhawk mounts (a light morph) at the starting point of the line. All four mounts were in a flight/attack position and had similar dimensions.


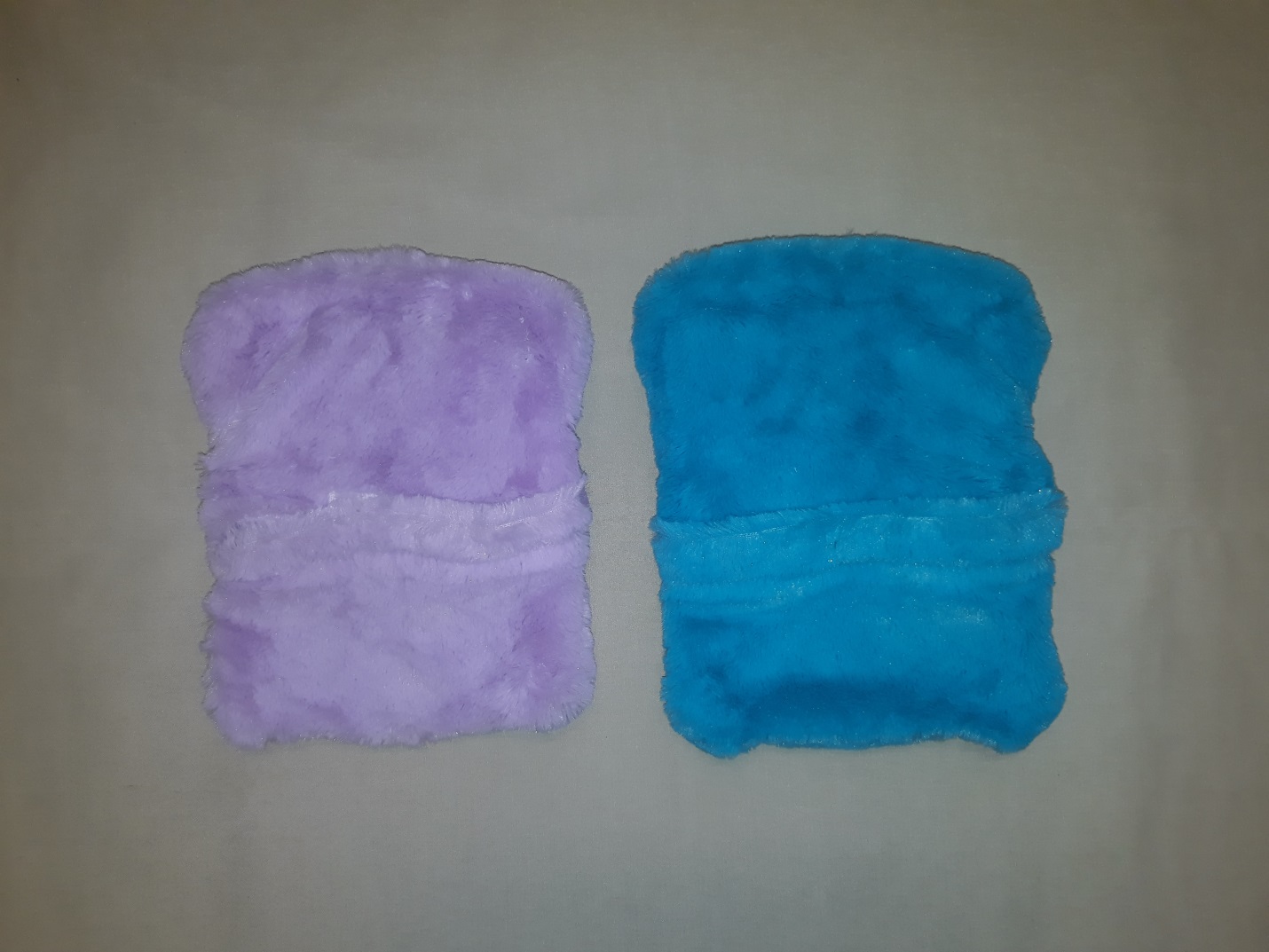


Figure S2. The two control objects flown instead of hawk mounts to test whether pigeons differentiated a control object from a predator attack. Control objects were 20 x 16 cm in size. We used two differently coloured controls, the pink fabric bag (left) and the blue fabric bag (right).

Table S1 lists illuminance levels (lux) measured in natural settings as part of this study. Ambient light levels were measured with a luxmeter (MS8229, Mastech). Respective date is given. “Type of environment” relates to the type of canopy (i.e. no tree canopy indicates an open environment with no trees providing shade, light forest a more open canopy and strong forest a more closed canopy). The “weather condition” briefly describes the weather when the measurement was taken and “time” the time of the day. “Lux” gives the illuminance measurements obtained; a range indicates that multiple measurements under slightly varying conditions were taken.

| **Date** | **Type of environment** | **Weather condition** | **Time** | **Lux** |
| --- | --- | --- | --- | --- |
| 24.4.2018 | No tree canopy | No overcast, direct sunlight | 10:00 am | Higher than 40 000 lux |
| 24.4.2018 | Light forest canopy | No overcast | 10:00 am | 2000 – 5000 lux |
| 26.4.2018 | Open, no tree canopy | Overcast, rain | 10:00 am | 3000 lux |
| 26.4.2018 | Light forest canopy | Overcast, heavy rain | 5:00 pm | 300 lux |
| 28.6.2018 | Open, no tree canopy | Overcast, no rain | 4:00 pm | 2000 – 2500 lux |
| 28.6.2018 | Strong forest canopy | Overcast, no rain | 4:00 pm | 125 - 250 lux |
| 28.6.2018 | Light forest canopy | Overcast, no rain | 4:00 pm | 100 – 1300 lux |
| 28.6.2018 | Open, no tree canopy | Overcast, no rain | 6:00 pm | 180 lux |
| 28.6.2018 | Strong forest canopy | Overcast, no rain | 6:00 pm | 12 lux |
| 28.6.2018 | Light forest canopy | Overcast, no rain | 6:00 pm | 25 lux |
| 29.6.2018 | Open, no tree canopy | Overcast, no rain | 7:30 am | 6 lux |
| 29.6.2018 | Open, no tree canopy | Overcast, no rain | 8:00 am | 100 lux |
| 29.6.2018 | Open, no tree canopy | Overcast, no rain | 8:30 am | 400 lux |

*Contrast ratio between hawk morphs and backgrounds*

We used the online tool based on the Web Content Accessibility Guidelines 2.0 (see contrast-ratio and WCAG10) to calculate the contrast ratio between the background and hawk colour in the RGB colour space, defined as the relative luminance (L). The highest ratio is obtained by plain black and plain white (L = 22) whereas a minimum score is reached by the same colours (L = 1). A high ratio therefore implies high colour contrast (and good visibility) whereas a low ratio indicates low colour contrast (better crypsis).

In our background experiment, we encounter two different light-background conditions: (1) low light – white background and (2) low light – black background. In the light-change experiment, we encounter two different light-background conditions; we used (1) bright light – white background (2) low light – white background

First, we evaluated the consistency in space and time of the background colouration. It showed a high consistency where the hawk first came into view, therefore we chose one pixel of the background colouration at the beginning of every trial video as the background colour.

Second, the contrast of the hawk against the background was measured at four fixed points: first, the breast (one pixel at the front, one in the back) and, second, the underwing coverts (left and right side). These four points are representing areas of high plumage colouration differences between the morphs. Here we expect to see the differences of the contrast ratio between morphs to show a difference with light and dark morphs having a high contrast ratio against a black or white background, respectively, and low contrast values where the colour of the hawk matches the colour of the background.

Table S2 shows the mean contrast ratio (relative luminance, L) and its standard deviation of the two black sparrowhawk morphs (dark or light) against the background colouration under varying conditions during this experiment. For the light-change experiment, attacks were simulated either under bright light – white background or low light – white background. In the background-change experiment, attacks were simulated either under low light – white background or low light – black background.

|  | Morph | |
| --- | --- | --- |
| Conditions | Light | Dark |
| Bright light – White background | 1.35 (SD 0.33) | 7.56 (SD 2.16) |
| Low light – White background | 1.55 (SD 0.34) | 2.8 (SD 0.09) |
| Low light – Black background | 5.49 (SD 2.22) | 1.20 (SD 0.10) |


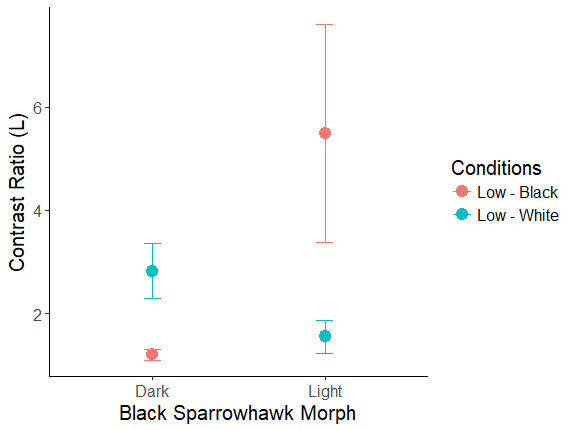


Figure S3 contrast ratio of the ventral colouration of the light and dark black sparrowhawk morph against the background colour in the background-change experiment. The conditions are Low – Black: low light – black background and Low – White: low light and white background. solid circles depict the contrast ratio (L) mean, error bars depict standard deviation. Mean and standard deviation were calculated based on four ventral point measurements per hawk (in total 20 measurements, ten per morph, four per hawk replicate).


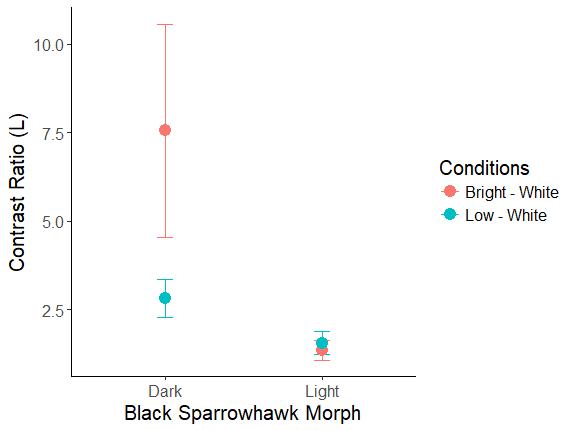


Figure S4 contrast ratio of the ventral colouration of the light and dark black sparrowhawk morph against the background colour in the light-change experiment. The conditions are Bright – White: bright light – white background and Low – White: low light and white background. Solid circles depict the contrast ratio (L) mean, error bars depict standard deviation. Mean and standard deviation were calculated based on four ventral point measurements per hawk (in total 20 measurements, ten per morph, four per hawk replicate).

The result of the contrast ratio shows a high consistency between trials for the two colour morphs. These results validate our methodology to measure contrast in the RGB colour space.

The ventral side shows varying contrast ratios, depending on the background colour and the morph of the attacking hawk. The light morph shows a high contrast ratio when attacking in front of a black background, similarly we obtained high contrast ratio for a dark morph attacking in front of a white background. Low contrast ratio values were recorded for the dark morph attacking in front of a black background and for a light morph attacking in front of a white background (Table S2, Figure S3).

No such effect was found for the light-change experiment – where the background colour stayed the same and only the light condition was altered. The contrast ratio measurements show that the contrast is very high for the dark morph under bright light levels but evens out and becomes more similar to the contrast ratios of the light morph when the light level is decreased. No large drop of the contrast ratio is observed for the light morph, likely because the background colour and the colour of the hawk mount were similarly affected by a change of light conditions (Table S2, Figure S4).”

REFERENCES

Online tool contrast-ratio: [www.contrast-ratio.com](http://www.contrast-ratio.com) (accessed 28.6.2019, 14:00)

WCAG10: Web Content Accessibility Guidelines 1.0 available at [www.w3.org/TR/WAI-WEBCONTENT/](http://www.w3.org/TR/WAI-WEBCONTENT/) (accessed 28.6.2019, 14:00)


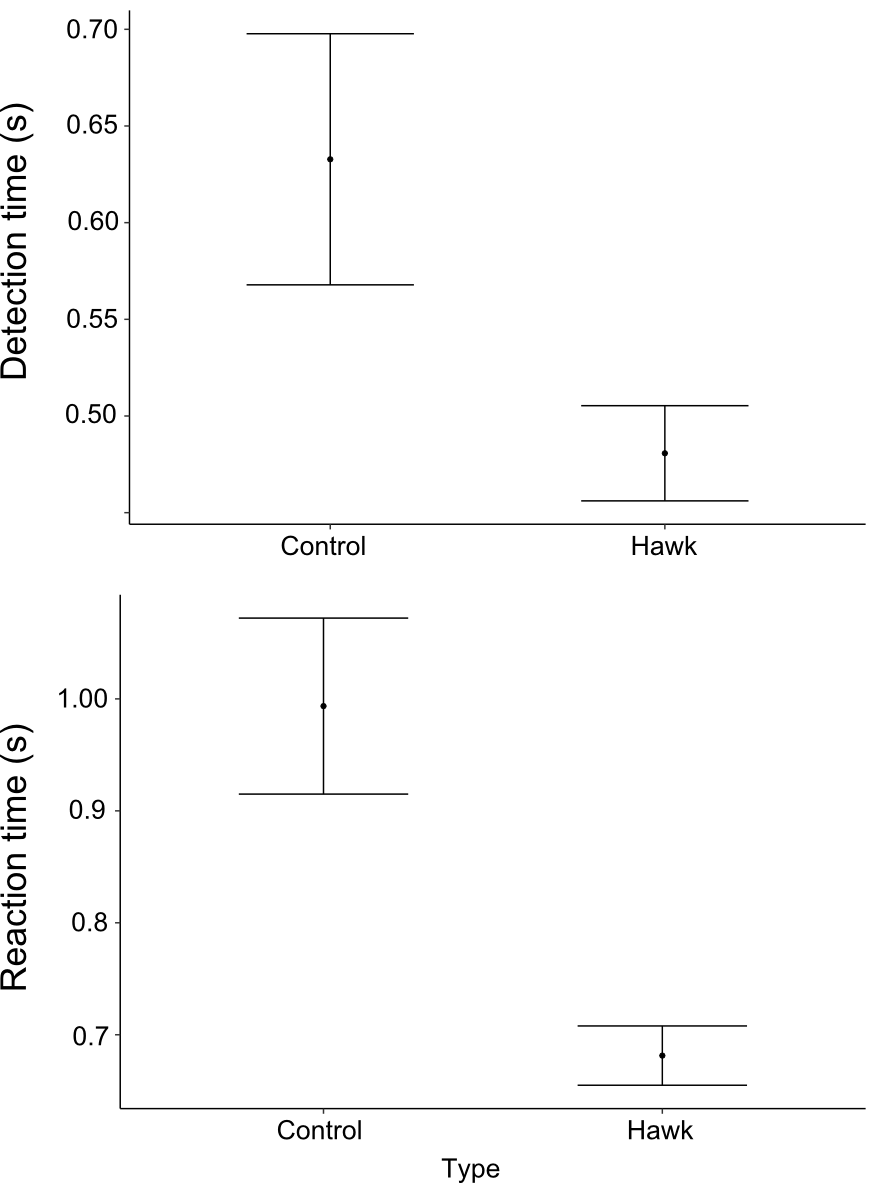


Figure S5. (a) Detection (top) and (b) reaction time (bottom) of pigeons to controls and hawks (attacks of black sparrowhawk mounts). Figure based on fitted values of LMMs with 95 % CIs.


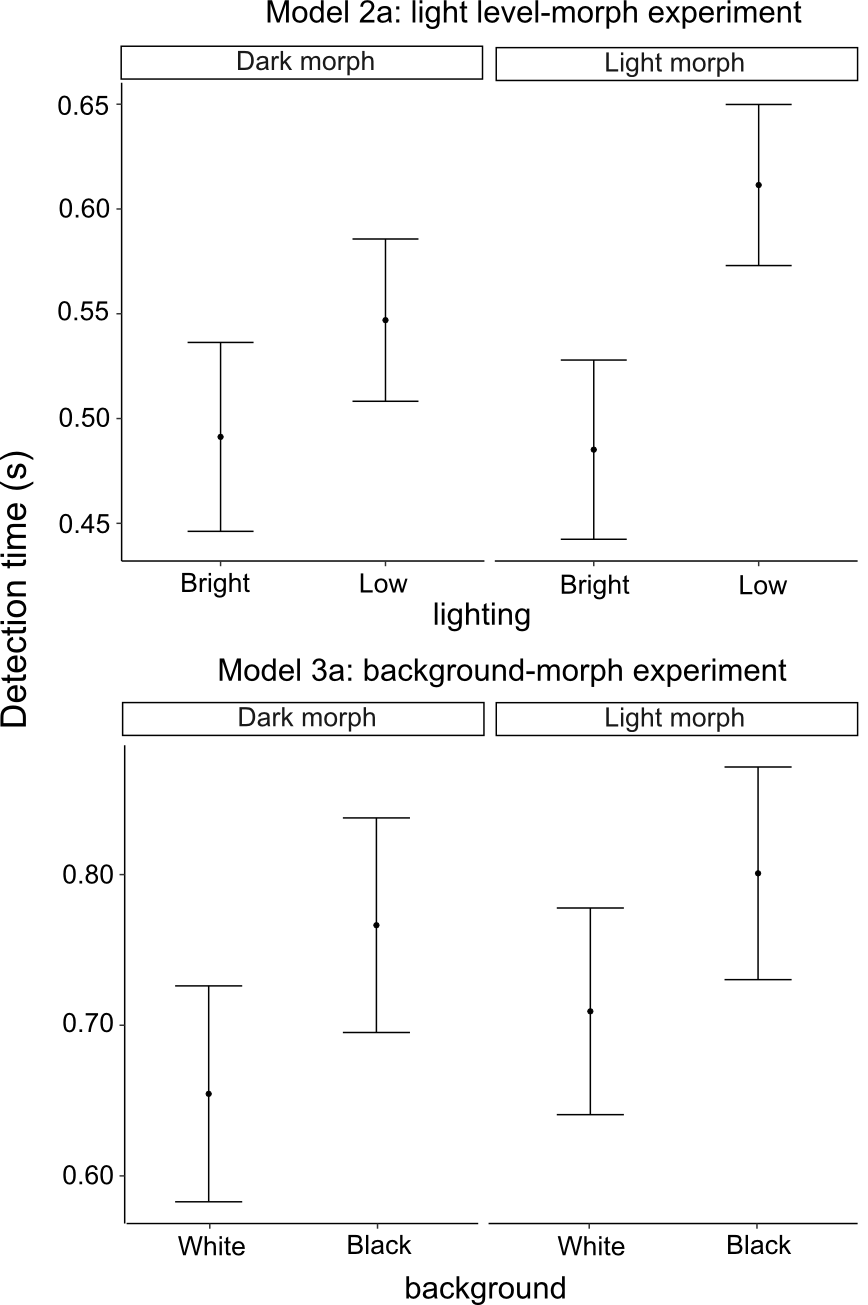


Figure S6. Detection time of pigeons to simulated attacks of black sparrowhawk mounts depending on (Model 2a) lighting: bright or dull, (Model 3a) background: black or white background. Figure based on fitted values of LMMs with 95 % CIs.

**A**


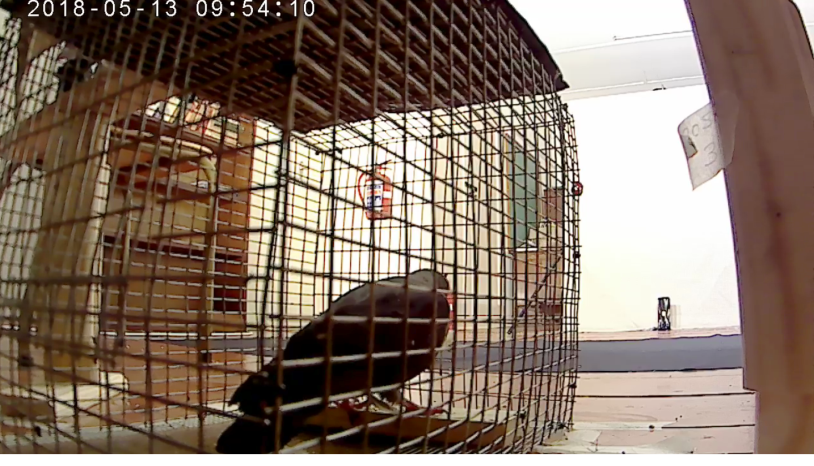


**B**


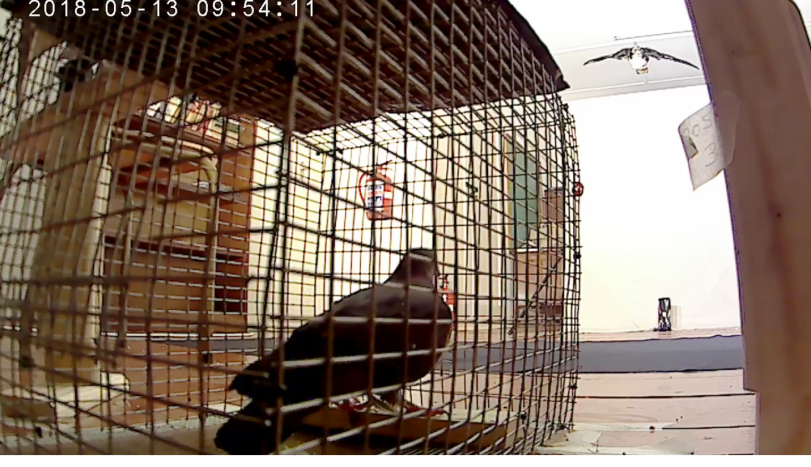


**C**


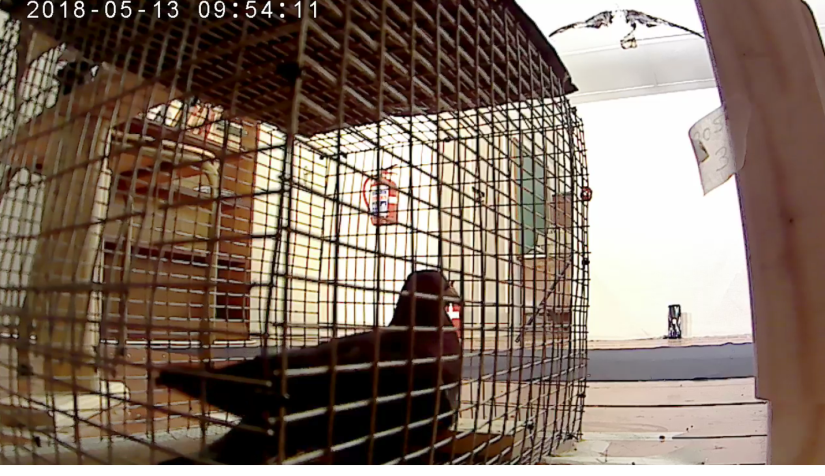


Figure S7. Example of a trial (with a white background, bright light levels and a light morph black sparrowhawk) with the three measuring points: In A, the pigeon is seen feeding during the trial and the hawk is barely visible over the blind. B shows the detection of the hawk by the pigeon (by raising its head from the feeder) and C is the initiation of an escape response (flight by foot into the back of the cage). The full video of the trial has been uploaded as Supplementary Material.
